# Quadruplex MAPH: improvement of throughput in high-resolution copy number screening

**DOI:** 10.1186/1471-2164-10-453

**Published:** 2009-09-28

**Authors:** Jess Tyson, Tamsin MO Majerus, Susan Walker, John AL Armour

**Affiliations:** 1 Institute of Genetics, School of Biology, University of Nottingham, Queen's Medical Centre, Nottingham NG7 2UH, UK

## Abstract

**Background:**

Copy number variation (CNV) in the human genome is recognised as a widespread and important source of human genetic variation. Now the challenge is to screen for these CNVs at high resolution in a reliable, accurate and cost-effective way.

**Results:**

Multiplex Amplifiable Probe Hybridisation (MAPH) is a sensitive, high-resolution technology appropriate for screening for CNVs in a defined region, for a targeted population. We have developed MAPH to a highly multiplexed format ("QuadMAPH") that allows the user a four-fold increase in the number of loci tested simultaneously. We have used this method to analyse a genomic region of 210 kb, including the *MSH2 *gene and 120 kb of flanking DNA. We show that the QuadMAPH probes report copy number with equivalent accuracy to simplex MAPH, reliably demonstrating diploid copy number in control samples and accurately detecting deletions in Hereditary Non-Polyposis Colorectal Cancer (HNPCC) samples.

**Conclusion:**

QuadMAPH is an accurate, high-resolution method that allows targeted screening of large numbers of subjects without the expense of genome-wide approaches. Whilst we have applied this technique to a region of the human genome, it is equally applicable to the genomes of other organisms.

## Background

Recent work has described the abundance of copy number variants (CNVs) in the human genome [1-4]. Not only have these studies demonstrated the presence of CNVs in apparently healthy individuals, and hence their considerable contribution to overall genetic variation, they have also shown CNVs to be an important contributor to susceptibility to complex disease [5-10].

A variety of different methods are available for the detection of genomic copy number changes [reviewed in [11,12]] each with their own advantages and disadvantages with respect to resolution, accuracy, applicability to large-scale studies and cost. Methods for copy number measurement can generally be divided into two broad categories: those that are suitable for global detection of novel variants (for example, array CGH and paired-end sequencing) as illustrated by [1,3,13,14], and more targeted methods that measure copy number at a particular locus, or relatively small number of loci, in a targeted population of probands (for example real time quantitative PCR, MAPH, MLPA and PRT) [15-18].

Multiplex Amplifiable Probe Hybridisation (MAPH) is a versatile and simple semi-quantitative method for direct determination of DNA copy number [16,19]. The method relies on the fact that amplifiable probes can be hybridised to genomic DNA fixed onto a nylon membrane, stringently washed, and then amplified so that the amount of amplified product is directly proportional to the copy number in the genomic DNA. Each amplifiable probe is a different length so that the probes can be resolved by electrophoresis. MAPH probes are also insensitive to substitutional polymorphisms making them highly suitable for copy number measurement. In addition, MAPH's multiplicity and flexible resolution means it is a method highly suited to accurate high resolution copy number screening.

To date, copy number measurement by MAPH has utilised a single set of probes, consisting of specifically designed amplicons cloned into a single vector, that measure the relative copy number of up to 40 targets simultaneously [20,21]. In order to screen for potential copy number changes at a greater number of loci simultaneously, we have introduced a novel scheme for probe generation, and developed the MAPH technology to use probe sets in a four-fold multiplex, here termed "QuadMAPH". This format has been particularly designed to allow thorough copy-number screening of 100-200 kb regions (for example, encompassing the genomic extent of a medium-sized human gene), at high (1-2 kb) resolution in large numbers of genomic DNA samples from selected cohorts. In this report we describe the development of a QuadMAPH screening method applied to a genomic region of over 200 kb around the *MSH2 *gene.

## Results

To generate a large number of MAPH probes within a specified region, restriction enzyme digestion of isolated BAC DNA was carried out to produce hundreds of fragments spanning a range of sizes. The individual combination of enzymes was selected of the basis of computer generated restriction enzyme prediction from the BAC sequence (macro (Microsoft Word) available on request). The digest simulation was used not only to predict the proportion of fragments that would be usable as MAPH probes but also the location of the usable probes with respect to BAC sequence to ensure a comprehensive coverage of the region of interest. The *Alu*I, *Hae*III, *Rsa*I digest simulation of BAC RP11-1084A21 predicted that from a total of 2243 (digest) fragments, 221 would be usable. Of the unusable fragments, 1336 were considered to be too short (<80 bp) and 13 too long (>500 bp). Among the 894 fragments in the 80-500 bp range, 149 were too AT rich (GC<35%) and 584 contained more than 20 bp of match to a masked repeat.

**Figure 1:**
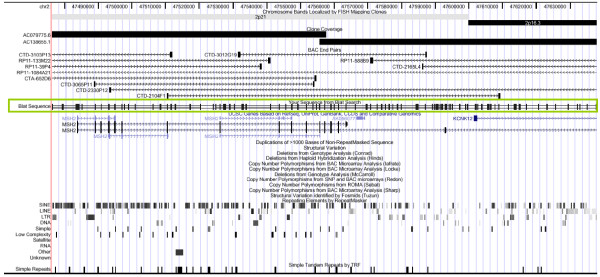
**Distribution of probes produced using a random cloning approach for *MSH2 *QuadMAPH set 1**. The window shows 210 kb DNA corresponding to BAC RP11-1084A21 and the location of the *MSH2 *gene on the UCSC genome browser [27,28] (March 2006 assembly). The positions of the probes are represented by the vertical black lines within the green box.

Following cloning, 864 colonies were picked from across the four vector systems and subjected to sequencing and bioinformatic analysis. 455 fragments satisfied the criteria for being usable MAPH probes, i.e., single copy, 80 to ≈500 base pairs in length and a GC content of 35% or above. The fragments ranged in actual size from 86 to 520 bp, and 37 to 62% GC. Following removal of duplicates in which the same fragment had been cloned more than once (in the same vector or between several vectors), 168 unique probes were accepted. Probe sets were assembled, taking into consideration coverage of the *MSH2 *region, GC content and range of probe sizes resolvable by capillary electrophoresis. Three control probes cloned into pZero2 were also included in the set. These consisted of X and Y linked probes and a non-human probe. Of the 168 unique MAPH probes for *MSH2 *generated by this method, 107 probes were assembled into an initial QuadMAPH set to screen the *MSH2 *locus at an average resolution of one probe per 1.96 kb. Distribution of *MSH2 *probes and coverage of the region from the initial QuadMAPH set is illustrated in Figure 1.

**Figure 2:**
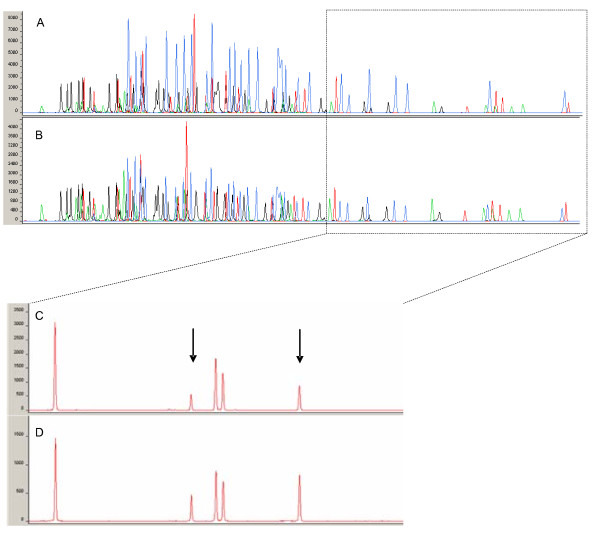
**A combined four-colour QuadMAPH trace for (A) an individual heterozygous for exonic deletions of the *MSH2 *gene and (B) a control**. Profiles from the pZero2, pGemT, pBS and XpUC probe sets are illustrated as blue (FAM), red (ROX), black (NED) and green (HEX) peaks respectively. The dashed box indicates the zoomed-in area illustrated in panels (C) and (D). Probes indicating a heterozygous deletion are shown by the arrows.

To assess the ability of QuadMAPH to identify deletion or duplication events, and to evaluate the measurement accuracy of the assay, a direct comparison was made between the HNPCC probe set [21] and our QuadMAPH set. MAPH using both probe sets was performed for 96 control DNA samples and 6 DNA samples with known *MSH2 *exonic deletions, which had been independently characterised by the referring laboratory. Example QuadMAPH traces for an individual heterozygous for exonic deletions of the *MSH2 *gene and a control are illustrated in Figure 2.

Analysis was carried out as previously described [20]. Briefly, experimental values are normalised relative to other probe results to give a normalised ratio (NR). The normalised ratios can be used as an index of the relative intensity of a given peak in a given sample, and hence be used to detect copy number changes. In the absence of copy number changes, a mean of 1 is expected, equivalent to a dosage of two copies per cell. A heterozygous deletion and a heterozygous duplication are expected to result in values of 0.5 and 1.5 respectively. Normalised ratios from control samples approximated well to a normal distribution. The accuracy of measurement by each probe was evaluated by calculating the standard deviation (SD) of the normalised ratio. The mean and SD of the NR values obtained for probes demonstrating diploid dosage and heterozygous deletion are shown in Table 1. Although individual probes differed in measurement accuracy, the average standard deviation of measurements, as illustrated in Table 1, was 0.0613 for HNPCC MAPH and 0.0671 for QuadMAPH. Assuming a normal (Gaussian) error distribution, this predicts a per test false positive rate of 9.9 × 10^-7 ^for HNPCC MAPH and 7.8 × 10^-6 ^for QuadMAPH at a threshold of 1 +/- 0.3. For deletion events, we predict a per test false negative rate of 1.5 × 10^-4 ^and 7.2 × 10^-4 ^for HNPCC and QuadMAPH respectively. For duplication events, we predict a per test false negative rate of 5.5 × 10^-4 ^and 1.4 × 10^-3 ^for HNPCC and QuadMAPH respectively.

**Table 1:** Mean normalised ratio (NR) and standard deviation (SD) of probes showing diploid dosage and those known to be involved in a heterozygous deletion.

	**Control probes**		**Probes within deletion**	
	**NR**	**SD**	**NR**	**SD**

Probe set: HNPCC	1.0005* (n = 3360)	0.0613	0.4952 (n = 61)	0.0552

Probe set: QuadMAPH	1.0003* (n = 9995)	0.0671	0.5986 (n = 58)	0.0628

*Initial normalisation of all the data included samples which were subsequently scored as deleted for a particular probe, leading to these control samples having an average value that is very slightly greater than 1.

## Discussion

The use of MAPH in assessing copy number changes at new loci requires the generation of new probe sets, which can be a lengthy and time-consuming process if individual amplicons need to be designed and cloned. Since many of the most likely candidates for disease association are in regions of the genome that also contain repeat elements, the positioning of single-copy probes can be problematic. Therefore efficient screening of regions for copy number variants by MAPH has necessitated the development of QuadMAPH and with it, the introduction of new cloning systems and a new method of generating probes.

Previously, MAPH probes have been made by designing and cloning individual PCR amplicons [20-22], which is not always appropriate for covering large genomic regions, or if numerous probes are required. In order to screen regions of approximately 200 kb in size with a probe approximately every 1-2 kb, we have established an alternative approach for MAPH probe generation, by which sets of probes are prepared by shotgun cloning of random short fragments from a BAC clone covering the region of interest. This method of producing MAPH probes is both efficient and cost-effective with respect to the number of probes generated and can be easily and rapidly applied to new loci which are not covered by commercially available technologies. An important step in the random generation of fragments is the choice of restriction enzymes in the initial BAC DNA digest, which dictates the number of usable MAPH probes produced. The use of double stranded linkers allows simultaneous PCR amplification of all fragments generated by this digestion. Digested BAC DNA was subject to two rounds of size-selection, which resulted in a reasonable distribution of fragments in the size range suitable for MAPH probes and was also necessary to offset any potential bias towards small fragments in the subsequent cloning and transformation steps.

Since the clones were generated randomly, unsurprisingly there was a degree of redundancy of fragments between vectors, but this allowed flexibility in the choice of probes for each set, and also produced a reserve of probes that could be used to improve on resolution or to confirm any potential copy number changes. In fact, once a set of clones has been generated, these can be stored in their 96-well microtitre plates at -20°C to -80°C thus providing a collection from which to make additional MAPH probe sets with minimal additional labour. Despite the high density of repetitive elements (SINEs) at the *MSH2 *locus, this approach generated good probe coverage across the region, as illustrated in Figure 1. If probe density is not sufficient to interrogate a specific region, then gaps can be filled by custom design of a small number of supplementary probes. Custom probes can be cloned into any one of four vectors to fit into size gaps in the profile for the vector chosen.

All four vectors can be multiplexed in any combination or used in MAPH on their own. Therefore, using four cloning systems allows the generation of four independent MAPH probe sets and subsequently a substantial increase in the number of targets that can be interrogated in a single test. The number of loci that can be analysed at once is determined by the number of probes in the probe set. Theoretically, the number of probes per set is only limited by the resolution and the number of fluorophores detectable after capillary electrophoresis, and in the case of generating probes by a random shotgun cloning approach, the size range of probes produced in the initial digest. Whilst our initial *MSH2 *QuadMAPH set consisted of 110 probes, additional probes can be included. In this work, by choosing an initial copy number screening resolution of a probe every 1-2 kb, the boundaries of any copy number variants discovered could then be defined within the range of long PCR, and junction-fragment analysis can be used to establish a rapid, specific PCR assay for the variant.

MAPH has previously been demonstrated as an accurate and precise assay for measurement of copy number [20-22]. After extensive experimental work dissecting and analysing factors affecting MAPH's accuracy and robustness, improvements have been made in the working method. PCR cycling modifications, and adherence to a strict wash protocol and wash solutions appropriate to the %GC content of the probe set, have reduced copy number measurement error and improved the reliability and reproducibility of the assay. As a consequence of this, the standard deviations reported here are considerably smaller than those reported by Hollox *et al *[20]. Associated with this is a substantial reduction in the predicted incidence of false positive and false negative results. The incorporation of control probes within a probe set, such as X and Y linked probes and a non-human probe, allow the user to assess non-specific probe binding and adjust washing conditions as necessary, as well as confirm that any copy number changes can be detected accurately based on the responsiveness of the control probes.

By comparison of data obtained by simplex MAPH with those by QuadMAPH, we have also demonstrated that multiplexing four probe sets is achieved with no ensuing loss of measurement accuracy, as indicated by the false positive and false negative predictions. Although MLPA has been successfully used in a two-colour format [23,24] these studies examined totals of 28 and 45 probes respectively, and we are not aware of successful use of MLPA at probe multiplicities approaching those in this report. Since MLPA also depends on pairwise ligation of half-probes, there may be a very large number of opportunities for MLPA using a large number of probes and multiple primers to create artefactual products; by contrast, MAPH probes in different vectors are not expected to interact with one another, and we have shown here that 110 probes perform well together. In summary, we believe QuadMAPH has the capacity to screen large numbers of loci simultaneously on a large number of samples.

## Conclusion

Genome wide studies have revealed the extent of copy number variation, but detailed investigation of specific regions creates a different challenge. At the present time, obtaining copy number variation information at the nucleotide level, on a genome-wide scale, in hundreds or thousands of individuals is not a realistic prospect for many investigators. For those who wish to examine copy number variation of a defined region(s) (of, for example, 100-200 kb) our four vector systems, combined with QuadMAPH, constitute an accurate, high-resolution method that allows targeted screening of large numbers of subjects without the expense of genome-wide approaches.

## Methods

### Preparation of BAC DNA linkered library

BAC DNA from RP11-1084A21, a clone obtained from BACPAC resources [25] covering the *MSH2 *gene and flanking regions, was prepared by alkaline lysis purification and 250 ng digested to completion with enzymes *Alu*I, *Hae*III, *Rsa*I. A fill-in step with 2.5 U Klenow polymerase and 1 μl 2.5 mM dNTPs was carried out at room temperature (20-25°C) for 1 hour, before purification by phenol/chloroform extraction and ethanol precipitation and ligation of fragments with double-stranded linkers. Linkers AH1 and AH2, incorporating restriction enzyme sites for *Alu*I and *Hae*III, were prepared at a final concentration of 500 μM dsDNA linker and 0.1 M NaCl by mixing equal volumes of 1 mM of each oligo AH1F (ACTGTCCAGCTTCGATGGCC) with AH1R (GGCCATCGAAGCTGGACA) (to generate linker AH1) and AH2F (CTAATCGGCCTGTGAGAGCT) with AH2R (AGCTCTCACAGGCCGATT) (for linker AH2) in the presence of 0.1 M NaCl and incubating at 65°C for 20 min followed by slow cooling. 25 ng BAC DNA digest fragments were then ligated at room temperature overnight with 100 μM AH1 and AH2 and 1 U T4 DNA ligase in a buffer containing 50 mM Tris-HCl, 10 mM MgCl_2_, 10 mM Dithiothreitol and 1 mM ATP. Linkered fragments were purified using the QIAquick PCR purification kit (Qiagen) according to the manufacturer's instructions, eluting in 50 μl EB buffer.

**Figure 3:**
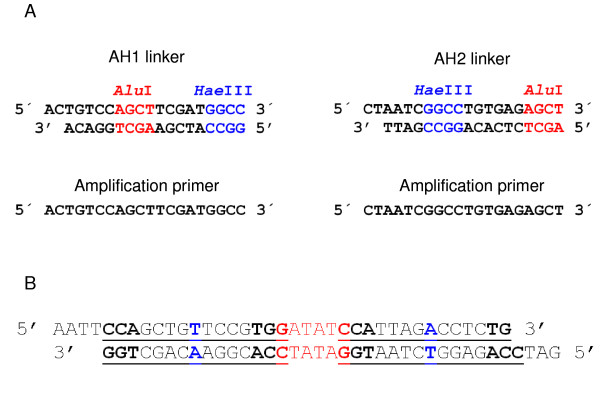
**Sequences of the double-stranded linkers (A) ligated to the digested fragments and the primers used for universal amplification, and (B) inserted into the pUC19 vector to create the XpUC vector**. The central *Eco*RV site is shown in red and the flanking recognition sequences for the two *Xcm*I sites highlighted bold. The base used to form the 3' T overhang is shown in blue.

In order to generate a sufficient quantity of fragments for subsequent size-selection, 2.5 μl of linkered BAC digest fragments were used to seed 8 separate 50 μl PCR reactions. Products were amplified using primers AH1F and AH2F under conditions of 70°C for 1 min, followed by 30 cycles of 95°C for 1 min, 60°C for 1 min, 70°C for 1 min. The PCR products were then pooled and purified using the QIAquick PCR purification kit (Qiagen), eluting in 30 μl EB. The sequences of the linkers and primers used to amplify the fragments are shown in Figure 3A.

### Size-selection of linkered fragments and preparation for cloning

Fragments were saturated with ethidium bromide before electrophoresis and separated on 1.5% agarose gel at 100V for precisely 90 min. A gel slice containing fragments in the size range 100 to 600 base pairs was excised using a Dark Reader Transilluminator. The gel slice was placed into a fresh agarose gel in an inverted orientation and the electrophoresis repeated at 100V for precisely 90 min to collect all fragments from the gel slice back into a single band. DNA was extracted from the gel using a QIAquick gel extraction kit (Qiagen) eluting in 30 μl EB buffer. Linker sequences were removed by restriction enzyme digestion with *Alu*I and *Hae*III and dephosphorylation carried out by addition of 1 U Calf Intestinal Alkaline Phosphatase for the final hour of digestion. A proportion of fragments were 3' dA tailed by incubating at 72°C for 1 hour with 1× BufferIV (ABgene), 1.6 mM MgCl_2_, 0.2 mM dATP and 1U *Taq *Polymerase to enable ligation into T-vector systems. All fragments were then size selected for a second time, as above.

### Vector preparation

In addition to the established plasmid vectors used for cloning MAPH probes, QuadMAPH required the introduction of two further vectors, pBS and XpUC. The T-vector pBS was prepared by restriction enzyme digestion of pBluescript II SK+ plasmid DNA (Stratagene) with *Eco*RV, followed by incubation with dideoxythymidine triphosphate (ddTTP) and terminal transferase [26] to create a single 3' T overhang.

An additional customised T-vector, XpUC, was prepared from a modified pUC19 plasmid by digestion with *Xcm*I. A linker was designed that contained an *Eco*RV site flanked by two *Xcm*I restriction enzyme sites. Each *Xcm*I site was specifically designed to have a thymine in the fifth 5' to 3' unspecified base position, such that a 3' T overhang would be created following digestion. The sequence of the double stranded linker is shown in Figure 3B. The linker fragment was inserted into the pUC19 plasmid between the *Eco*RI and *Bam*HI sites of the polylinker. To aid purification of the final vector, a 1 kb 'stuffer' fragment from ΦX174 DNA-*Hae*III digest was ligated into the *Eco*RV site at the centre of the linker. XpUC vector was prepared from this modified plasmid by digestion with *Sac*II (to linearise the plasmid via a site in the 'stuffer' fragment) and subsequently *Xcm*I. To limit the potential for recircularisation or dimer formation during ligation, a self-ligation step followed by selection of the unligated linear vector from an agarose gel was carried out.

### Cloning and transformation

Approximately 50-100 ng of size-selected DNA fragments, in a volume of 2 μl, were ligated with 25 ng of one of four different plasmid vectors, pZero2 [Invitrogen, see [16]], pGemT (Promega), pBS and XpUC, and purified by ethanol precipitation prior to transformation. Transformation using 2 μl ligation reaction per 25 μl *E. coli *Top 10 cells (Invitrogen) was carried out by electroporation at 12.5 kV/cm (200Ω, 25 μF). Cells were incubated in 500 μl SOC for one hour at 37°C, shaking to mix. 25 μl 1 M IPTG was added for cloning into pGemT and pBS. Cells were spread on LB agar plates containing either 50 μg/ml Kanamycin for pZero2 or 100 μg/ml Ampicillin for pGemT, pBS and XpUC and incubated at 37°C overnight. For pGemT and pBS 25 μl of XGal (25 mg/ml) was added to each plate before spreading to allow blue/white screening. Following overnight incubation at 37°C, colonies were picked into individual wells of 96-well microtitre plates containing 100 μl LB broth, 4% glycerol and the appropriate antibiotic and incubated at 37°C overnight.

### Identification of usable MAPH probes

Inserts from recombinant plasmids were amplified by PCR directly from the 96-well microtitre plate cultures using primer 1277 (TGGCGAAAGGGGGATGTGCTG) common to all vectors and a vector specific primer (PZA for pZero2, PGB for pGemT, PBSA for pBS or PUCB for XpUC clones; Table 2), with 25 cycles of 95°C for 30 s, 60°C for 1 min and 70°C for 1 min. PCR products were purified using AMPure (Agencourt) and 20-30 ng sequenced with primer 1277 using 0.25 μl BigDye Terminator v3.1 mix and 3.75 μl 5× BigDye sequencing buffer (Applied Biosystems) using standard cycle sequencing conditions. Sequenced products were purified using CleanSEQ (Agencourt) following the manufacturer's protocol.

**Table 2:** Sequences of all MAPH primers used in this study.

**Primer name**	**Sequence**
Fam PZA	Fam-AGTAACGGCCGCCAGTGTGCTG

PZB	CGAGCGGCCGCCAGTGTGATG

Rox PGA	Rox-CCGCCATGGCCGCGGGAT

PGB	AGGCGGCCGCACTAGTGAT

Ned-PBSB	Ned-GGTCGACGGTATCGATAAGCTTG

PBSA	CCCCCGGGCTGCAGGAATTC

PBSA2	GCCCCCGGGCTGCAGGAATTC

Hex-PUCB	Hex-CGACTCTAGAGGATCCAGAGG

PUCA	CGACGGCCAGTGAATTCCAG

The sequences of all cloned fragments were subject to bioinformatic analysis to identify those fragments that satisfied criteria for being usable MAPH probes. All insert sequences were aligned with the human genome using the BLAT search facility at UCSC (March 2006 assembly [27,28]). Any inserts which failed to match the source BAC or contained a perfect match of over 30 bases to another genomic location were rejected. Size and GC content were also taken into consideration. Finally, the remaining probes were aligned with the BAC sequence using align 2 sequences blast facility at NCBI [29]. This allowed the precise location of each insert within the region to be recorded. We will be happy to make the custom bioinformatic tools used freely available to interested researchers.

Plasmid DNA from selected clones was isolated using the R.E.A.L. Prep 96 plasmid procedure (Qiagen) according to the manufacturer's instructions, with the exception that all inversion steps were replaced by gentle mixing by pipette to reduce potential well-to-well contamination of plasmid DNA. MAPH probes were amplified from purified plasmid DNA using vector specific primers: PZA with PZB for pZero2; PGA with PGB for pGemT; PBSA2 with PBSB for pBS, and PUCA with PUCB for XpUC. The sequences of all primers are shown in Table 2. Amplification was carried out for 20 cycles of 95°C for 1 min, 60°C for 1 min and 70°C for 1 min. Product concentration was estimated on an agarose gel and probes mixed in vector specific sets to give a final concentration of 2 ng/μl per probe following purification by QIAquick PCR purification kit (Qiagen).

### MAPH

UK control samples used in this study consisted of ECACC Human Random control (HRC) panels 1 and 2 [30]. HNPCC disease cohort DNA was kindly supplied by Wessex Regional Genetics Laboratory. MAPH was carried out according to [16,20] with the following modifications. During the hybridisation step, *E. coli/Hae*III DNA (at 3.5 mg/ml) was omitted. For QuadMAPH, hybridisation of the four independent probe sets occurred simultaneously in the same hybridisation tube, with additional 20 pmol of each of the 'end-blocking' primers PGA, PGB, PBSA, PBSB, PUCA and PUCB. Washing to remove unbound probes was carried out at 65°C using wash solutions of 1 × SSC, 1%SDS for solution 1 and 0.1 × SSC, 0.1%SDS for solution 2. Washed filters were transferred to 50 μl 1× buffer IV (ABgene) and heated at 95°C for 5 min. 1 μl of this was used to seed subsequent PCR reactions. For MAPH utilising a single probe set, PCR was carried out using Fam-PZA with PZB for 25 cycles of 95°C for 30 s, 60°C for 1 min, 70°C for 1 min, followed by a final extension at 70°C for 40 min. 1 μl of PCR product was mixed with 10 μl HiDi formamide and ROX500 marker (Applied Biosystems). PCR to amplify the recovered probes in QuadMAPH was achieved in two duplex PCR reactions using primers FAM-PZA and PZB in a reaction with ROX-PGA and PGB, and NED-PBSB and PBSA2 with HEX-PUCB and PUCA, using PCR conditions detailed above. 1 μl of each duplex PCR product was mixed with 10 μl HiDi formamide (Applied Biosystems). All samples were denatured at 96°C for 3 min and then placed on ice prior to electrophoresis on an ABI 3100 36 cm capillary, using POP-4 polymer with an injection time of 45 s. Peaks were quantified using Genescan and Genotyper software (Applied Biosystems) using a MAPH trace as an internal size standard in place of ROX500 for the QuadMAPH data. Data analysis using Excel (Microsoft) was carried out as previously described [20]. For QuadMAPH, each independent probe set was analysed separately. Full details of macros, probes and probe sets used are available on request.

## Authors' contributions

JT, TM and SW did the experiments and data analysis; JT and TM took the lead in writing the paper; JA conceived, designed and directed the study, as well as making a contribution to experimental work. All authors contributed to the final manuscript.
